# *N*-{4-[(4-Methyl­phen­yl)sulfamo­yl]phen­yl}acetamide

**DOI:** 10.1107/S160053681002739X

**Published:** 2010-07-17

**Authors:** Peter John, Islam Ullah Khan, Muhammad Arif Sajjad, Shahzad Sharif, Edward R. T. Tiekink

**Affiliations:** a Materials Chemistry Laboratory, Department of Chemistry, Government College University, Lahore 54000, Pakistan; b Department of Chemistry, University of Malaya, 50603 Kuala Lumpur, Malaysia

## Abstract

The title mol­ecule, C_15_H_16_N_2_O_3_S, has a twisted U-shaped conformation. The twist occurs in the central C—S(=O)_2_N(H)—C unit with the C—S—N—C torsion angle being −58.38 (14)°. The benzene rings lie to the same side of the mol­ecule and form a dihedral angle of 67.03 (10)°. The crystal packing features N—H⋯O hydrogen bonding, which leads to supra­molecular chains with a tubular topology along the *b* axis. Intra­molecular C—H⋯O inter­actions are also observed.

## Related literature

For background to the pharmacological uses of sulfonamides, see: Beate *et al.* (1998 ▶); Kazmierski *et al.* (2004 ▶). For related structures, see: Khan *et al.* (2010 ▶); Sharif *et al.* (2010 ▶).
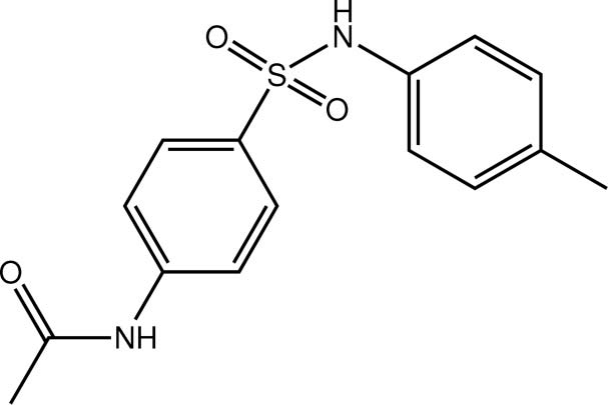


## Experimental

### 

#### Crystal data

C_15_H_16_N_2_O_3_S
*M* *_r_* = 304.37
Triclinic, 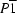

*a* = 8.2224 (3) Å
*b* = 8.3878 (3) Å
*c* = 13.0796 (5) Å
α = 71.482 (2)°
β = 75.749 (2)°
γ = 61.265 (1)°
*V* = 745.27 (5) Å^3^
*Z* = 2
Mo *K*α radiation
μ = 0.23 mm^−1^
*T* = 293 K
0.23 × 0.14 × 0.08 mm

#### Data collection

Bruker APEXII CCD diffractometer
Absorption correction: multi-scan (*SADABS*; Sheldrick, 1996 ▶) *T*_min_ = 0.887, *T*_max_ = 0.951
12704 measured reflections
3374 independent reflections
2918 reflections with *I* > 2σ(*I*)
*R*_int_ = 0.024

#### Refinement

*R*[*F* ^2^ > 2σ(*F* ^2^)] = 0.041
*wR*(*F* ^2^) = 0.124
*S* = 1.04
3374 reflections
198 parameters
2 restraints
H atoms treated by a mixture of independent and constrained refinement
Δρ_max_ = 0.34 e Å^−3^
Δρ_min_ = −0.34 e Å^−3^

### 

Data collection: *APEX2* (Bruker, 2007 ▶); cell refinement: *SAINT* (Bruker, 2007 ▶); data reduction: *SAINT*; program(s) used to solve structure: *SHELXS97* (Sheldrick, 2008 ▶); program(s) used to refine structure: *SHELXL97* (Sheldrick, 2008 ▶); molecular graphics: *ORTEP-3* (Farrugia, 1997 ▶) and *DIAMOND* (Brandenburg, 2006 ▶); software used to prepare material for publication: *publCIF* (Westrip, 2010 ▶).

## Supplementary Material

Crystal structure: contains datablocks global, I. DOI: 10.1107/S160053681002739X/pv2305sup1.cif

Structure factors: contains datablocks I. DOI: 10.1107/S160053681002739X/pv2305Isup2.hkl

Additional supplementary materials:  crystallographic information; 3D view; checkCIF report

## Figures and Tables

**Table 1:** Hydrogen-bond geometry (Å, °)

*D*—H⋯*A*	*D*—H	H⋯*A*	*D*⋯*A*	*D*—H⋯*A*
C6—H6⋯O1	0.93	2.60	3.125 (3)	116
C10—H10⋯O3	0.93	2.22	2.818 (3)	121
C13—H13⋯O1	0.93	2.56	2.914 (3)	103
N1—H1n⋯O3^i^	0.88 (2)	1.99 (2)	2.853 (3)	169 (2)
N2—H2n⋯O2^ii^	0.87 (2)	2.18 (2)	3.029 (2)	165 (2)

Symmetry codes: (i) 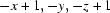; (ii) 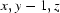.

**Fig. 1.**
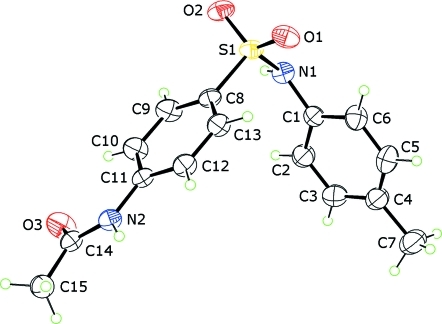
The molecular structure of (I) showing the atom-labelling scheme and displacement ellipsoids at the 35% probability level.

**Fig. 2.**
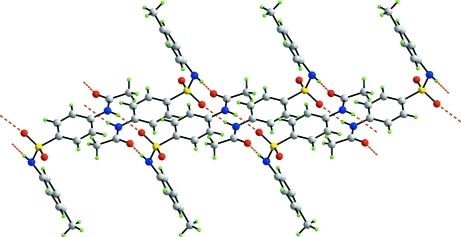
A view of the supramolecular double chain along the b axis in (I) mediated by N–H···O hydrogen bonding (orange dashed lines) in (I). Colour code: S, yellow; O, red; N, blue; C, grey; and H, green.

**Fig. 3.**
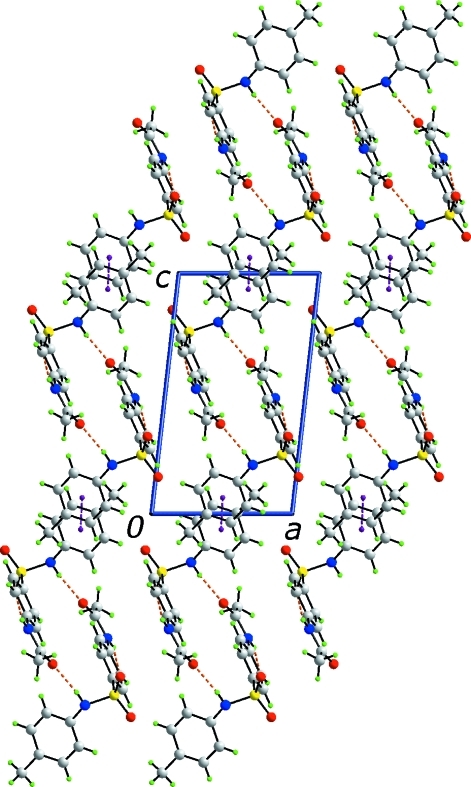
A view in projection down the a axis of the crystal packing in (I), highlighting the tubular topology of the double chains (shown in Fig. 2) and the π···π interactions (purple dashed lines).

## supplementary crystallographic information

### Comment

Sulfonamide drugs are used, for example, as inhibitors of HIV infection (Kazmierski *et al.*, 2004) and as anti-hypertensive drugs (Beate *et al.*, 1998). In connection with on-going structural studies of sulfonamides (Khan *et al.*, 2010; Sharif *et al.*, 2010), the crystal and molecular structure of the title compound, (I), was investigated.

To a first approximation, the molecule of (I), Fig. 1, has a twisted U-shaped conformation with the two benzene residues projecting to the same side of the molecule with reference to the central S(═O)_2_N moiety but, being splayed with respect to each other. The dihedral angle formed between the two benzene rings is 67.03 (10) ° and the C1–N1–S1–C8 torsion angle of -58.38 (14) ° indicates a twist in the molecule. Both S-bound O atoms lie to one side of the S-bound benzene ring [the O1–S1–C8–C9 torsion angle = 169.12 (13) ° and O2–S1–C8–C9 = 39.25 (15) °], and the N1 atom to the other [N1–S1–C8–C9 = -73.19 (15) °]. The amide group is co-planar with the benzene ring to which it is attached as seen in the C10–C11—N2–C14 torsion angle of 7.2 (3) °.

The formation of N–H···O hydrogen bonds dominate the crystal packing, Table 1. Thus, the sulfamoyl-N1–H hydrogen bonds to the amide-O, and the amide-N2–H hydrogen bonds to a sulfamoyl-O2 atom; the sulfamoyl-O1 atom forms intramolecular C–H···O interactions as does the amide-O3 atom, Table 1. The N–H···O hydrogen bonds result in the formation of a supramolecular chain along the *b* axis with a tubular topology, Fig. 2. These assemble into layers in the *ab* plane. Interactions between layers are of the type π···π [ring centroid(C1–C6)···ring centroid(C1–C6)^i^ = 3.8185 (13) for *i*: 1 - *x*, -*y*, 2 - *z*], Fig. 3.

### Experimental

A mixture of 4-methylaniline (250 mg, 2.33 mmol) and 4-(acetylamino)benzenesulfonyl chloride (545 mg, 2.33 mmol) in distilled water (25 ml) was stirred, while maintaining pH of the reaction mixture at 8–10 by adding 3% sodium carbonate solution. The reaction was monitored by TLC. After completion of the reaction, precipitates were filtered, washed and dried. The crude product obtained was crystallized by dissolving in methanol followed by slow evaporation of the solvent. *M*. pt. 493 K.

### Refinement

The C-bound H atoms were geometrically placed (C–H = 0.93–0.96 Å) and refined as riding with *U_iso_*(H) = 1.2–1.5*U_eq_*(C). The N-bound H atoms were refined with the distance restraint N–H = 0.88±0.01 Å, and with *U_iso_*(H) = 1.2*U_eq_*(N). In the final refinement three low angle reflections evidently effected by the beam stop were omitted, *i.e.* 0 0 1, 0 1 0, and 0 1 1.

### Crystal data

**Table:**

C_15_H_16_N_2_O_3_S	*Z* = 2
*M**_r_* = 304.37	*F*(000) = 320
Triclinic, *P*1	*D*_x_ = 1.356 Mg m^−^^3^
Hall symbol: -P 1	Mo *K*α radiation, λ = 0.71073 Å
*a* = 8.2224 (3) Å	Cell parameters from 7070 reflections
*b* = 8.3878 (3) Å	θ = 2.9–28.3°
*c* = 13.0796 (5) Å	µ = 0.23 mm^−^^1^
α = 71.482 (2)°	*T* = 293 K
β = 75.749 (2)°	Block, colourless
γ = 61.265 (1)°	0.23 × 0.14 × 0.08 mm
*V* = 745.27 (5) Å^3^	

### Data collection

**Table:**

Bruker APEXII CCD diffractometer	3374 independent reflections
Radiation source: fine-focus sealed tube	2918 reflections with *I* > 2σ(*I*)
graphite	*R*_int_ = 0.024
φ and ω scans	θ_max_ = 27.5°, θ_min_ = 1.7°
Absorption correction: multi-scan (*SADABS*; Sheldrick, 1996)	*h* = −10→10
*T*_min_ = 0.887, *T*_max_ = 0.951	*k* = −10→10
12704 measured reflections	*l* = −16→16

### Refinement

**Table:**

Refinement on *F*^2^	Primary atom site location: structure-invariant direct methods
Least-squares matrix: full	Secondary atom site location: difference Fourier map
*R*[*F*^2^ > 2σ(*F*^2^)] = 0.041	Hydrogen site location: inferred from neighbouring sites
*wR*(*F*^2^) = 0.124	H atoms treated by a mixture of independent and constrained refinement
*S* = 1.04	*w* = 1/[σ^2^(*F*_o_^2^) + (0.0681*P*)^2^ + 0.1946*P*] where *P* = (*F*_o_^2^ + 2*F*_c_^2^)/3
3374 reflections	(Δ/σ)_max_ = 0.001
198 parameters	Δρ_max_ = 0.34 e Å^−^^3^
2 restraints	Δρ_min_ = −0.34 e Å^−^^3^

### Special details

**Table:**

Geometry. All s.u.'s (except the s.u. in the dihedral angle between two l.s. planes) are estimated using the full covariance matrix. The cell s.u.'s are taken into account individually in the estimation of s.u.'s in distances, angles and torsion angles; correlations between s.u.'s in cell parameters are only used when they are defined by crystal symmetry. An approximate (isotropic) treatment of cell s.u.'s is used for estimating s.u.'s involving l.s. planes.
Refinement. Refinement of *F*^2^ against ALL reflections. The weighted *R*-factor *wR* and goodness of fit *S* are based on *F*^2^, conventional *R*-factors *R* are based on *F*, with *F* set to zero for negative *F*^2^. The threshold expression of *F*^2^ > 2σ(*F*^2^) is used only for calculating *R*-factors(gt) *etc*. and is not relevant to the choice of reflections for refinement. *R*-factors based on *F*^2^ are statistically about twice as large as those based on *F*, and *R*- factors based on ALL data will be even larger.

### Fractional atomic coordinates and isotropic or equivalent isotropic displacement parameters (Å^2^)

**Table:**

	*x*	*y*	*z*	*U*_iso_*/*U*_eq_	
S1	0.12002 (5)	0.25013 (5)	0.75637 (3)	0.04453 (15)	
O1	−0.01522 (16)	0.24530 (17)	0.84756 (11)	0.0578 (3)	
O2	0.09188 (18)	0.42467 (16)	0.67943 (11)	0.0581 (3)	
O3	0.4166 (2)	−0.2526 (2)	0.37348 (12)	0.0728 (4)	
N1	0.31999 (19)	0.17594 (19)	0.79741 (11)	0.0459 (3)	
H1N	0.391 (2)	0.214 (3)	0.7440 (11)	0.055*	
N2	0.21546 (19)	−0.29178 (19)	0.51939 (11)	0.0437 (3)	
H2N	0.164 (2)	−0.365 (2)	0.5573 (13)	0.052*	
C1	0.4103 (2)	−0.0054 (2)	0.86613 (12)	0.0429 (3)	
C2	0.5858 (3)	−0.1276 (3)	0.83046 (15)	0.0614 (5)	
H2	0.6388	−0.0963	0.7601	0.074*	
C3	0.6828 (3)	−0.2962 (3)	0.89901 (17)	0.0693 (5)	
H3	0.8020	−0.3765	0.8746	0.083*	
C4	0.6068 (3)	−0.3481 (3)	1.00264 (16)	0.0596 (5)	
C5	0.4290 (3)	−0.2261 (3)	1.03546 (15)	0.0633 (5)	
H5	0.3738	−0.2601	1.1047	0.076*	
C6	0.3305 (3)	−0.0558 (3)	0.96922 (14)	0.0553 (4)	
H6	0.2113	0.0244	0.9938	0.066*	
C7	0.7165 (4)	−0.5295 (3)	1.0789 (2)	0.0835 (7)	
H7A	0.7837	−0.5081	1.1196	0.125*	
H7B	0.8030	−0.6197	1.0377	0.125*	
H7C	0.6324	−0.5759	1.1278	0.125*	
C8	0.1436 (2)	0.0924 (2)	0.68549 (13)	0.0405 (3)	
C9	0.2424 (3)	0.0930 (2)	0.58393 (15)	0.0541 (4)	
H9	0.2931	0.1780	0.5537	0.065*	
C10	0.2674 (3)	−0.0305 (2)	0.52644 (14)	0.0532 (4)	
H10	0.3330	−0.0276	0.4575	0.064*	
C11	0.1941 (2)	−0.1593 (2)	0.57195 (12)	0.0387 (3)	
C12	0.0926 (2)	−0.1576 (2)	0.67372 (13)	0.0468 (4)	
H12	0.0410	−0.2419	0.7040	0.056*	
C13	0.0671 (2)	−0.0332 (2)	0.73066 (13)	0.0472 (4)	
H13	−0.0010	−0.0335	0.7989	0.057*	
C14	0.3207 (2)	−0.3310 (2)	0.42545 (13)	0.0451 (4)	
C15	0.3142 (3)	−0.4801 (2)	0.38969 (15)	0.0561 (4)	
H15A	0.3141	−0.4457	0.3123	0.084*	
H15B	0.2028	−0.4941	0.4240	0.084*	
H15C	0.4215	−0.5962	0.4098	0.084*	

### Atomic displacement parameters (Å^2^)

**Table:**

	*U*^11^	*U*^22^	*U*^33^	*U*^12^	*U*^13^	*U*^23^
S1	0.0380 (2)	0.0362 (2)	0.0593 (3)	−0.01654 (16)	−0.00415 (16)	−0.01162 (17)
O1	0.0426 (6)	0.0572 (7)	0.0737 (8)	−0.0215 (5)	0.0084 (6)	−0.0273 (6)
O2	0.0592 (7)	0.0366 (6)	0.0783 (8)	−0.0216 (5)	−0.0167 (6)	−0.0052 (6)
O3	0.0807 (10)	0.0762 (9)	0.0703 (8)	−0.0511 (8)	0.0268 (7)	−0.0277 (7)
N1	0.0425 (7)	0.0476 (7)	0.0521 (7)	−0.0252 (6)	−0.0035 (6)	−0.0098 (6)
N2	0.0436 (7)	0.0411 (7)	0.0487 (7)	−0.0239 (6)	−0.0029 (5)	−0.0061 (5)
C1	0.0433 (8)	0.0455 (8)	0.0463 (8)	−0.0229 (7)	−0.0056 (6)	−0.0127 (6)
C2	0.0550 (10)	0.0598 (11)	0.0530 (9)	−0.0163 (9)	0.0022 (8)	−0.0132 (8)
C3	0.0625 (12)	0.0567 (11)	0.0703 (12)	−0.0092 (9)	−0.0084 (10)	−0.0185 (9)
C4	0.0733 (12)	0.0496 (10)	0.0644 (11)	−0.0289 (9)	−0.0261 (9)	−0.0068 (8)
C5	0.0692 (12)	0.0741 (13)	0.0490 (9)	−0.0402 (11)	−0.0099 (8)	−0.0003 (9)
C6	0.0471 (9)	0.0671 (11)	0.0497 (9)	−0.0262 (8)	−0.0002 (7)	−0.0130 (8)
C7	0.1059 (19)	0.0585 (13)	0.0869 (15)	−0.0302 (13)	−0.0458 (14)	−0.0009 (11)
C8	0.0349 (7)	0.0340 (7)	0.0512 (8)	−0.0156 (6)	−0.0072 (6)	−0.0056 (6)
C9	0.0579 (10)	0.0488 (9)	0.0631 (10)	−0.0371 (8)	0.0089 (8)	−0.0120 (8)
C10	0.0578 (10)	0.0520 (9)	0.0530 (9)	−0.0345 (8)	0.0110 (7)	−0.0124 (7)
C11	0.0333 (7)	0.0342 (7)	0.0455 (7)	−0.0151 (6)	−0.0079 (6)	−0.0022 (6)
C12	0.0528 (9)	0.0448 (8)	0.0483 (8)	−0.0322 (7)	0.0000 (7)	−0.0043 (7)
C13	0.0525 (9)	0.0460 (8)	0.0458 (8)	−0.0300 (7)	0.0017 (7)	−0.0060 (6)
C14	0.0395 (8)	0.0408 (8)	0.0474 (8)	−0.0137 (6)	−0.0080 (6)	−0.0048 (6)
C15	0.0551 (10)	0.0522 (10)	0.0601 (10)	−0.0186 (8)	−0.0121 (8)	−0.0155 (8)

### Geometric parameters (Å, °)

**Table:**

S1—O1	1.4214 (13)	C5—H5	0.9300
S1—O2	1.4362 (12)	C6—H6	0.9300
S1—N1	1.6178 (14)	C7—H7A	0.9600
S1—C8	1.7581 (16)	C7—H7B	0.9600
O3—C14	1.211 (2)	C7—H7C	0.9600
N1—C1	1.431 (2)	C8—C9	1.376 (2)
N1—H1N	0.877 (9)	C8—C13	1.383 (2)
N2—C14	1.351 (2)	C9—C10	1.376 (2)
N2—C11	1.406 (2)	C9—H9	0.9300
N2—H2N	0.867 (9)	C10—C11	1.388 (2)
C1—C2	1.378 (2)	C10—H10	0.9300
C1—C6	1.377 (2)	C11—C12	1.386 (2)
C2—C3	1.379 (3)	C12—C13	1.376 (2)
C2—H2	0.9300	C12—H12	0.9300
C3—C4	1.374 (3)	C13—H13	0.9300
C3—H3	0.9300	C14—C15	1.495 (2)
C4—C5	1.378 (3)	C15—H15A	0.9600
C4—C7	1.511 (3)	C15—H15B	0.9600
C5—C6	1.376 (3)	C15—H15C	0.9600
			
O1—S1—O2	119.51 (8)	H7A—C7—H7B	109.5
O1—S1—N1	109.54 (8)	C4—C7—H7C	109.5
O2—S1—N1	104.83 (7)	H7A—C7—H7C	109.5
O1—S1—C8	107.30 (7)	H7B—C7—H7C	109.5
O2—S1—C8	107.71 (8)	C9—C8—C13	119.74 (15)
N1—S1—C8	107.41 (7)	C9—C8—S1	119.07 (12)
C1—N1—S1	122.71 (10)	C13—C8—S1	121.19 (12)
C1—N1—H1N	116.6 (13)	C8—C9—C10	121.02 (14)
S1—N1—H1N	108.7 (13)	C8—C9—H9	119.5
C14—N2—C11	128.32 (13)	C10—C9—H9	119.5
C14—N2—H2N	117.5 (13)	C9—C10—C11	119.64 (15)
C11—N2—H2N	113.7 (12)	C9—C10—H10	120.2
C2—C1—C6	119.49 (17)	C11—C10—H10	120.2
C2—C1—N1	119.02 (15)	C12—C11—C10	119.07 (15)
C6—C1—N1	121.37 (15)	C12—C11—N2	117.67 (13)
C1—C2—C3	120.06 (18)	C10—C11—N2	123.25 (14)
C1—C2—H2	120.0	C13—C12—C11	121.06 (14)
C3—C2—H2	120.0	C13—C12—H12	119.5
C4—C3—C2	121.35 (19)	C11—C12—H12	119.5
C4—C3—H3	119.3	C12—C13—C8	119.45 (15)
C2—C3—H3	119.3	C12—C13—H13	120.3
C3—C4—C5	117.56 (18)	C8—C13—H13	120.3
C3—C4—C7	121.1 (2)	O3—C14—N2	122.59 (16)
C5—C4—C7	121.3 (2)	O3—C14—C15	121.55 (16)
C6—C5—C4	122.19 (18)	N2—C14—C15	115.85 (15)
C6—C5—H5	118.9	C14—C15—H15A	109.5
C4—C5—H5	118.9	C14—C15—H15B	109.5
C5—C6—C1	119.30 (17)	H15A—C15—H15B	109.5
C5—C6—H6	120.3	C14—C15—H15C	109.5
C1—C6—H6	120.3	H15A—C15—H15C	109.5
C4—C7—H7A	109.5	H15B—C15—H15C	109.5
C4—C7—H7B	109.5		
			
O1—S1—N1—C1	57.85 (14)	O1—S1—C8—C13	−11.81 (16)
O2—S1—N1—C1	−172.76 (12)	O2—S1—C8—C13	−141.68 (14)
C8—S1—N1—C1	−58.38 (14)	N1—S1—C8—C13	105.88 (14)
S1—N1—C1—C2	121.29 (16)	C13—C8—C9—C10	−0.3 (3)
S1—N1—C1—C6	−62.61 (19)	S1—C8—C9—C10	178.75 (15)
C6—C1—C2—C3	−2.2 (3)	C8—C9—C10—C11	−0.9 (3)
N1—C1—C2—C3	173.98 (18)	C9—C10—C11—C12	1.7 (3)
C1—C2—C3—C4	1.3 (3)	C9—C10—C11—N2	−179.09 (16)
C2—C3—C4—C5	0.6 (3)	C14—N2—C11—C12	−173.58 (15)
C2—C3—C4—C7	−177.6 (2)	C14—N2—C11—C10	7.2 (3)
C3—C4—C5—C6	−1.7 (3)	C10—C11—C12—C13	−1.3 (2)
C7—C4—C5—C6	176.52 (19)	N2—C11—C12—C13	179.41 (15)
C4—C5—C6—C1	0.8 (3)	C11—C12—C13—C8	0.1 (3)
C2—C1—C6—C5	1.2 (3)	C9—C8—C13—C12	0.7 (3)
N1—C1—C6—C5	−174.91 (16)	S1—C8—C13—C12	−178.36 (13)
O1—S1—C8—C9	169.12 (13)	C11—N2—C14—O3	1.1 (3)
O2—S1—C8—C9	39.25 (15)	C11—N2—C14—C15	−179.77 (14)
N1—S1—C8—C9	−73.19 (15)		

### Hydrogen-bond geometry (Å, °)

**Table:**

*D*—H···*A*	*D*—H	H···*A*	*D*···*A*	*D*—H···*A*
C6—H6···O1	0.93	2.60	3.125 (3)	116
C10—H10···O3	0.93	2.22	2.818 (3)	121
C13—H13···O1	0.93	2.56	2.914 (3)	103
N1—H1n···O3^i^	0.88 (2)	1.99 (2)	2.853 (3)	169 (2)
N2—H2n···O2^ii^	0.87 (2)	2.18 (2)	3.029 (2)	165 (2)

Symmetry codes:  (i) −*x*+1, −*y*, −*z*+1; (ii) *x*, *y*−1, *z*.
